# Presence of pathogen DNA in milk harvested from quarters is associated to changes in cows’ milk yield and composition

**DOI:** 10.1186/s12917-024-04083-y

**Published:** 2024-06-07

**Authors:** Silvia Magro, Elena Visentin, Angela Costa, Mauro Penasa, Filippo Cendron, Paolo Moroni, Elena Chiarin, Martino Cassandro, Matteo Santinello, Massimo De Marchi

**Affiliations:** 1 Department of Agronomy, Food, Natural resources, Animals and Environment, University of Padova, Legnaro, 35020 Italy; 2 Department of Veterinary Medical Sciences, Alma Mater Studiorum University of Bologna, Ozzano dell’Emilia, 40064 Italy; 3 Department of Veterinary Medicine and Animal Sciences, University of Milan, Lodi, 26900 Italy; 4 Laboratorio di Malattie Infettive degli Animali, University of Milan, Lodi, 26900 Italy; 5 Associazione Nazionale Allevatori della Razza Frisona, Bruna e Jersey Italiana, Cremona, 26100 Italy

**Keywords:** Intramammary infection, Sterile milk sampling, qPCR, Udder health

## Abstract

**Background:**

Intramammary infection is the result of invasion and multiplication of microorganisms in the mammary gland and commonly leads to mastitis in dairy animals. Although much has been done to improve cows’ udder health, mastitis remains a significant and costly health issue for dairy farmers, especially if subclinical. In this study, quarter milk samples from clinically healthy cows were harvested to detect pathogens via quantitative PCR (qPCR) and evaluate changes in individual milk traits according to the number of quarters infected and the type of microorganism(s). A commercial qPCR kit was used for detection of *Mycoplasma bovis*, *Mycoplasma* spp., *Staphylococcus aureus*, coagulase-negative staphylococci (CNS), *Streptococcus agalactiae*, *Streptococcus dysgalactiae*, *Streptococcus uberis*, *Prototheca* spp., *Escherichia coli*, *Klebsiella* spp., *Enterococcus* spp. and *Lactococcus lactis* ssp. *lactis.* Quarter and pooled milk information of 383 Holstein, 132 Simmental, 129 Rendena, and 112 Jersey cows in 9 Italian single-breed herds was available.

**Results:**

Among the cows with pathogen(s) present in at least 1 quarter, CNS was the most commonly detected DNA, followed by *Streptococcus uberis*, *Mycoplasma bovis*, and *Streptococcus agalactiae*. Cows negative to qPCR were 206 and had the lowest milk somatic cell count. Viceversa, cows with DNA isolated in ≥ 3 quarters were those with the highest somatic cell count. Moreover, when major pathogens were isolated in ≥ 3 quarters, milk had the lowest casein index and lactose content. In animals with pathogen(s) DNA isolated, the extent with whom milk yield and major solids were impaired did not significantly differ between major and minor pathogens.

**Conclusions:**

The effect of the number of affected quarters on the pool milk quality traits was investigated in clinically healthy cows using a commercial kit. Results remark the important negative effect of subclinical udder inflammations on milk yield and quality, but more efforts should be made to investigate the presence of untargeted microorganisms, as they may be potentially dangerous for cows. For a smarter use of antimicrobials, analysis of milk via qPCR is advisable – especially in cows at dry off - to identify quarters at high risk of inflammation and thus apply a targeted/tailored treatment.

## Background

The term intramammary infection (IMI) commonly refers to an invasion and multiplication of microorganisms in the alveoli and/or the ducts and tubules of the mammary gland [1]. In cows, IMI commonly results into clinical or subclinical mastitis which is still one of the most common and impacting diseases that occur in dairy farms [2]. The first line of defense against IMI is both anatomical and physiological, located in the teat end [3]. During and immediately after milking, the teat canal is dilated and vulnerable to penetration of mastitis-causing microorganisms which may activate leukocyte populations - specifically polymorphonuclear neutrophils (PMN) - for their antibacterial activity [4, 5]. To destroy the bacteria, PMN release oxidants and proteases that can damage the alveolar epithelium permeability [6]. Therefore, cows with IMI have high leukocytes (white blood cells) in their milk, i.e., high somatic cell count (SCC) and differential SCC (DSCC) [7], which is the percentage of PMN and lymphocytes out of the total SCC. The DSCC does not consider the macrophages fraction of SCC and authors suggested that it could be useful to identify certain types of mastitis [8–11].

Mastitis-causing agents can be divided into major and minor pathogens according to their prevalence and the severity of symptoms [12], but it is important to consider that clinically healthy cows, which also include those with a subclinical form of mastitis or with latent IMI, may present pathogens in their milk due to their presence in the cistern or contamination in the teat canal (end and orifice). However, if white blood cells are succesfull, presence of colonies does not necessarily translate into inflammation [13]. Knowing in advance if there are cases in the herd with mastitis-causing pathogen(s) DNA in milk could be useful for decision-making at farm level, especially for cows approaching the dry-off. Although the gold standard for qualitative and quantitative analysis of microorganisms is the bacterial culture, the quantitative PCR (qPCR) has been developed for faster and cheaper identification of known microorganism species [14], including pathogens that cannot be grown using conventional culturing techniques, such as *Mycoplasma* spp. [15]. Morevoer, the Matrix-Assisted Laser Desorption/Ionization – Time of Flight (MALDI-TOF) is nowadays recognized as an accurate method fot pathogens identification in various medium, including milk, but it cannot be adopted routinarily by commercial farmers for the costs [16]. Thus, the present study aims to evaluate the effect of the presence of pathogen(s) DNA on both milk yield and composition on sterile milk samples collected at quarter level in clinically healthy cows.

## Methods

### Samples collection

This study has been funded by the research project DOC-AR 2021 (“Dry-Off Cow and Antibiotic Reduction”) of the Breeders Association of Veneto Region (ARAV, Vicenza, Italy). The experimental design and the protocol were approved by ARAV and farmers that voluntarily joined the research via informed consent.

A total of 799 clinically healthy cows, i.e., nor with clinical mastitis in the lactation and in the previous lactation nor under any antimicrobial treatment, were involved. Animals were sampled between June and December 2021 and belonged to 9 single-breed herds with similar farming system (intensive) located in the Po Valley in the Veneto region: 4 Holstein, 2 Simmental, 1 Jersey, and 2 Rendena herds.

On the day of sampling, each cow’s quarter milk sample was collected in sterile conditions (STER), whereas 1 composite (pooled) milk sample was collected conventionally from the milkmeter in non-aseptic conditions by trained personell of ARAV, which is a branch of the Italian Breeders Association (AIA, Rome, Italy). Before the STER sampling, a normal pre-milking routine was adopted, including manual forestripping. Subsequently, according to National Mastitis Council guidelines [17], teat ends and orificies were cleaned with single disposable wet disinfectant towels (Kerbl, Buchbach, Germany). For the STER aliquots intended for qPCR, approximately 5 mL of milk from each quarter was aseptically collected in sterile tubes containing Bronopol (2-bromo-2-nitropropan-1,3-diol).

**Fig. 1:**
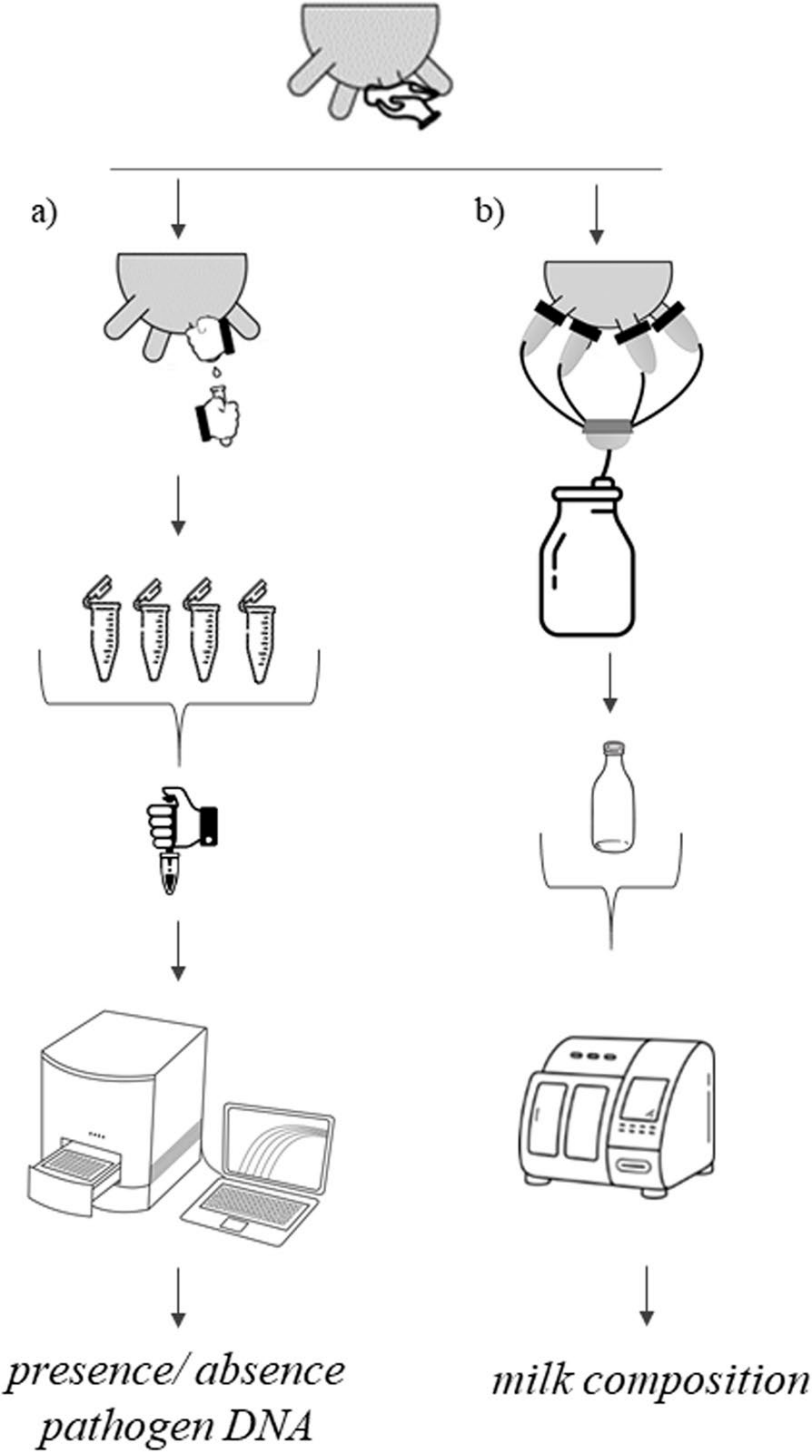
Overview of the milk sampling procedure^1^ adopted. ^1^**a** Quarter-level milk harvested for detection of pathogen DNA via qPCR (STER); **b** composite milk for composition traits assessment through infrared spectroscopy (DHI)

The STER aliquots containing quarter milk were transported to the milk laboratory of ARAV and stored at − 20 °C until the qPCR analysis, whereas the pooled milk was just refrigerated analysed within 48 h in the same laboratory. The composite milk sample used in this study, in fact, corresponded to the official one (50 mL) to be tested within the Italian Dairy Herd Improvement (DHI) framewrok (Fig. 1), i.e. refrigerated (4 °C) and processed with a CombiFoss™ 7 analyser (Foss Electric A/S, Hillerød, Denmark). Fat, protein, casein, and lactose content were determined via infrared spectroscopy and both SCC and DSCC via flow citometry. Information on DIM, parity, and daily milk yield was retrieved.

### qPCR

The commercial quantitative qPCR kit ‘Mastitis 4BDF’ (DNA Diagnostic A/S, Risskov, Denmark), already used in previous studies [18, 19], was chosen to isolate DNA of *Mycoplasma bovis (M. bovis), Mycoplasma* spp., *Staphylococcus aureus (S. aureus), Streptococcus agalactiae (S. agalactiae)*, coagulase-negative staphylococci (CNS), *Streptococcus dysgalactiae (S. dysgalactiae), Streptococcus uberis (S. uberis), Prototheca* spp., *Escherichia coli (E. coli), Klebsiella* spp. *(K. pneumonie, K. oxytoca, and K. variicola)* and *Enterococcus* spp. + *Lactococcus lactis* ssp. *lactis (L. lactis* spp. *lactis)* combined. Although currently it is common to talk about non-*aureus* staphylococci (NAS) instead of CNS, in this study, for consistency, the abbreviation CNS was used in accordance with the kits’ manufacturer.

After thawing and inversion of STER samples, a representative volume (0.50 mL) was used for total DNA extraction according to the instructions (DNA Diagnostic A/S, 2017). The qPCR reaction was performed in the AriaMx Real-Time PCR System (Agilent Technologies Inc., Santa Clara, CA) and run under the following amplification conditions: 95 °C for 1 min for 1 cycle, and 95 °C for 5 s and 60 °C for 25 s for 40 cycles. The assay’s protocol involved three separate multiplex qPCR reactions, each of which targeted four pathogens and an internal amplification control. Cycle threshold values were reported for all samples. For all the pathogens identified in the analysis, a cycle threshold value ≤ 37 was considered a positive result and samples with greater values were considered as free from pathogen DNA [18].

### Statistical analysis

Data manipulation, editing, and analysis were carried out using various packages of the R software v. 4.1.3 [20]. The qPCR results were dichotomized for each pathogen DNA as either presence or absence by using the cut-off cycle threshold value (≤ 37) [21]. Quarter milk samples were considered as contaminated when 3 or more different target pathogens DNA were identified [22]. Cows with at least 1 contaminated quarter/STER sample were excluded from statistical analysis (*n* = 43), leading to a final dataset of 756 cows in 9 herds, all with information on presence/absence of pathogen DNA at quarter level and pooled milk yield and gross composition. All cows involved had 4 functional teats, i.e., 4 STER samples.

A cow was considered as infected when at least one of the target pathogens was isolated in at least one quarter. Conversely, animals were considered as not infected when all the quarters were free from pathogens, i.e., no pathogen DNA was detected within cow. Nevertheless, it is important to highlight that the kit is species-specific [21], therefore it cannot be excluded that third pathogens - not identifiable by the ‘Mastitis 4BDF’ kit - could be present in the sampled quarters.

For the statistical analysis, pathogens were grouped and classes were created based on the number of quarters presenting pathogen DNA (≤ 2 or ≥ 3 quarters). In line with Kirkeby et al. [19] and Schwarz et al. [23], whenever present, the pathogens were categorized as follows:

- major (*S. agalactiae*, *S. aureus*, *S. uberis* and *S. dysgalactiae*, *E. coli*, and *Klebsiella* spp.);
- minor (CNS);
- other (*M. bovis*, *Mycoplasma* spp., *Prothoteca*, and *Enterococcus* spp.*+ L. lactis* ssp. *lactis*).

Table 1 reports the number of cows in each pathogen group and number of quarters with pathogen DNA. Some animals presented different pathogen DNA (in the same quarter or in different quarters), so, for instance, cows in the ‘other pathogen’ group could have minor pathogens too (Table 1).

**Table 1:** Number of cows in each pathogen group

Group	Level	Cows, *n*	Minor pathogens included^a^			Other pathogens included^b^		
			0	≤ 2	≥ 3	0	≤ 2	≥ 3
Absent pathogens^c^		206						
Major pathogens^c^	≤ 2 quarters	248	111	84	53	209	27	12
	≥ 3 quarters	31	16	9	6	28	2	1
Minor pathogens^c^	≤ 2 quarters	151						
	≥ 3 quarters	62						
Other pathogens^c^	≤ 2 quarters	42	18	16	8			
	≥ 3 quarters	16	10	5	1			

^a^N. cows that had minor pathogens in addition to major or other pathogens

^b^N. cows that had other pathogens in addition to major pathogens

^c^Pathogens of the commercial kit 'Mastitis 4BDF'. CNS indicates the coagulase-negative staphylococci

Values of milk yield, and contents of fat, protein, casein, and lactose deviating more than 3 standard deviations from the respective mean were considered as outliers and removed. Casein index was calculated as the ratio of casein to protein content and, starting from the SCC and DSCC available, the number of PMN and lymphocytes excreted (DSCC_N_, cells/mL) were calculated as reported in Costa et al. [24]: DSCC_N_ = (SCC × DSCC)/100. To make SCC data points normally distributed, the score was obtained through the formula of Ali and Shook [25]: SCS = 3 + log_2_(SCC/100,000). For the same purpose, DSCC_N_ was log_2_-transformed and the differential somatic cell score (DSCS) was obtained. Pearson’s correlations were calculated and analysis of variance was performed considering milk yield and composition traits as dependent variables. The first model (Eq. 1) was useful to compare udder health traits of quarters free from pathogens vs. infected; the second one (Eq. 2) was needed to compare milk yield and compositon of different pathogens groups:

1 $$y_{ijklm}\;=\;\mu\;+\;P_i\;+\;D_j\;+\;DNA_k\;+\;B_l\;+\;H_m(B_l)\;+\;e_{ijklm}$$

2 $$y_{ijklm}\;=\;\mu\;+\;P_i\;+\;D_j\;+\;Pathogens_k\;+\;B_l\;+\;H_m(B_l)\;+\;e_{ijklm}$$

where *y*_*ijklm*_ is the dependent variable; µ is the overall intercept of the model; *P*_*i*_ is the fixed effect of the *i*th parity (*i* = 1, 2, ≥ 3, with the last class including parity up to 11); *D*_*j*_ is the fixed effect of the *j*th stage of lactation (*j* = 8 classes, with the first being a class from 5 to 50 DIM, followed by 6 classes of 50 DIM each, and the last being a class from 351 to 900 DIM); *DNA*_*k*_ is the fixed effect of *k*th class of pathogen (presence/absence); *Pathogens*_*k*_ is the fixed effect of the *k*th pathogen group (*k* = absent, ‘minor’ ≤ 2 quarters, ‘minor’ ≥ 3 quarters, ‘major’ ≤ 2 quarters, ‘major’ ≥ 3 quarters, ‘other’ ≤ 2 quarters, and ‘other’ ≥ 3 quarters); *B*_*l*_ is the fixed effect of the *l*th breed (*l* = Holstein, Simmental, Rendena, Jersey); *H*_*m*_ is the fixed effect of the *m*th herd (*m* = 1 to 9) nested within the *l*th breed; and *e*_*ijklm*_ is the random error. Model diagnostics were checked through analysis of distribution, variance homogeneity, and independence of residuals. Multiple comparisons of least squares means (LSM) were performed using the Bonferroni adjustment with significance set at *P* ≤ 0.05. Contrast estimates between LSM of fixed effects included in Eq. 2 were used to compare specific groups of pathogens (absent vs. present, minor, major or other, minor vs. major, minor vs. other and major vs. other; Table 1). In particular, the following groups were used:

- major pathogens (2 levels): cows with ≤ or > 2 quarters with DNA isolated;
- minor pathogens (2 levels): cows with ≤ or > 2 quarters with DNA isolated;
- other pathogens (2 levels): cows with ≤ or > 2 quarters with DNA isolated.

## Results

### Overview

Milk was sampled from the four functional quarters of 799 cows. 43 cows were excluded from the analysis due to presence of 1 or more contaminated milk samples. Of the remaining 756 cows, i.e., 383 Holstein, 132 Simmental, 129 Rendena, and 112 Jersey (Table 2), 206 were negative to the test, as no DNA of the target pathogens (‘Mastitis 4BDF’ kit) was detected. In this qualitative commercial kit, whenever a pathogen DNA was isolated, it was not possible to retrieve the specific location, i.e., it cannot be excluded that the DNA originated from the teat canal (infected) and not from a quarter with an ongoing infection.

**Table 2:** General characteristics^a^ of the 9 herds

Characteristic		Herd								
		1	2	3	4	5	6	7	8	9
	Breed	Holstein	Holstein	Holstein	Holstein	Jersey	Rendena	Rendena	Simmental	Simmental
	Cows, n	76	77	123	107	112	89	40	101	31
	DIM, d	156 (135)	174 (115)	353 (130)	251 (108)	176 (132)	229 (140)	234 (111)	195 (96)	167 (111)
	Parity	2.46 (1.77)	2.29 (1.21)	2.93 (2.17)	1.96 (0.94)	1.80 (0.92)	3.11 (2.09)	3.35 (2.43)	2.90 (1.73)	2.84 (1.59)
	Milk yield, kg/d	32.52 (9.98)	34.06 (8.38)	20.66 (8.62)	31.67 (6.57)	23.48 (6.95)	16.41 (6.04)	23.86 (5.92)	31.54 (7.86)	25.16 (6.01)
	SCC, cells/mL	41,500	80,000	102,000	55,000	85,000	166,000	65,000	48,000	93,000
Major^b^	*S. agalactiae*	✔	✔		✔	✔	✔	✔	✔	
	*S. uberis*	✔	✔	✔	✔	✔	✔	✔	✔	✔
	*S. dysagalactiae*	✔	✔	✔	✔	✔	✔	✔	✔	✔
	*S. aureus*		✔	✔	✔	✔			✔	✔
	*E. coli*	✔	✔	✔	✔	✔	✔	✔		
	*Klebsiella* spp.	✔	✔	✔		✔		✔	✔	
Minor^b^	CNS	✔	✔	✔	✔	✔	✔	✔	✔	✔
Other^b^	*M. bovis*	✔	✔	✔	✔	✔	✔	✔	✔	
	*Mycoplasma* spp.		✔	✔			✔		✔	✔
	*Enterococcus + L. lactis* ssp. *Lactis*			✔	✔	✔	✔	✔	✔	
	*Prototheca* spp.			✔	✔		✔			

^a^Mean (standard deviation) for DIM, parity, and milk yield, median for SCC, and presence (✔) of pathogens DNA in at least one cow. P

^b^Pathogens of the commercial kit 'Mastitis 4BDF'. CNS indicates the coagulase-negative staphylococci

**Fig. 2:**
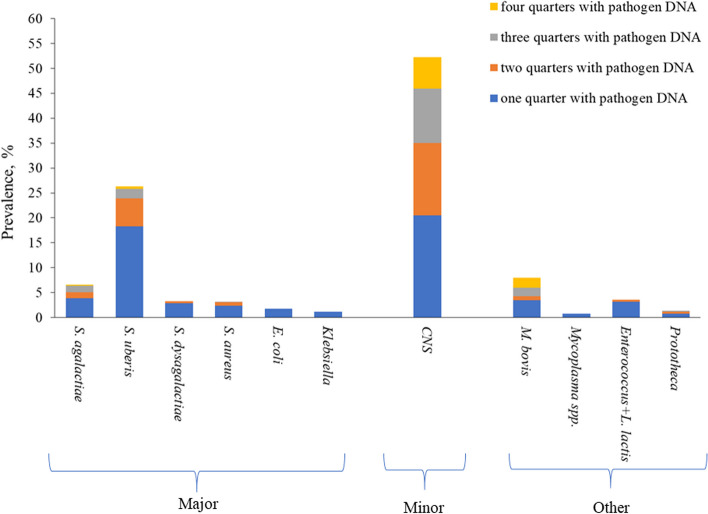
Prevalence of each pathogen DNA (3,014 quarter milk samples of 756 cows)

Among the cows positive to the qPCR, CNS was the most commonly detected species followed by *S. uberis*, *M. bovis*, and *S. agalactiae*. In fact, in this study CNS belonged to the minor pathogen group and were isolated in 52.2% of the animals (Fig. 2), with an average of 2.1 positive quarters/cow (Table 3).

**Table 3:** Distribution and prevalence of pathogen DNA^a^ isolated from 3,014 quarter milk samples of 756 cows

Group	Pathogen	At quarter level (*n* = 3,014)		At cow level^b^(*n* = 756)		Average *n*. affected quarters^c^
		*n*	%	*n*	%	
Major	*S. agalactiae*	85	2.8	50	6.6	1.7
	*S. uberis*	282	9.4	199	26.3	1.4
	*S. dysgalactiae*	28	0.9	25	3.3	1.1
	*S. aureus*	31	1.0	24	3.2	1.3
	*E. coli*	13	0.4	13	1.7	1.0
	*Klebsiella* spp.	9	0.3	9	1.2	1.0
Minor	CNS	813	27.0	395	52.2	2.1
Other	*M. bovis*	137	4.5	60	7.9	2.8
	*Mycoplasma* spp.	6	0.2	6	0.8	1.0
	*Enterococcus + Lactococcus lactis* ssp. *lactis*	30	1.0	27	3.6	1.1
	*Prototheca* spp.	15	0.5	10	1.3	1.5

^a^Pathogens of the commercial kit 'Mastitis 4BDF'. CNS indicates the coagulase-negative staphylococci

^b^Pathogen DNA isolated in at least 1 quarter

^c^Quarters per cow with pathogen DNA detected

Regarding major pathogens, *S. uberis* and *S. agalactiae* were isolated in 26.3% and 6.6% of cows (Table 3), most having DNA present in only 1 quarter. *S. dysgalactiae* and *S. aureus* were detected in 25 and 24 cows, respectively, with an average of 1.1 (*S. dysgalactiae*) and 1.3 (*S. aureus*) quarters with DNA of these pathogens (Table 3). DNA of *E. coli* and *Klebsiella* spp. was isolated in a small number of cows (13 and 9 out of 756, respectively) and only in one quarter (Table 3; Fig. 2).

As regards the ‘other pathogen’ group, *M. bovis* was detected in 7.9% of cows, with an average of 2.8 quarters (Fig. 2; Table 3). *Prototheca* spp. DNA was isolated in 1.3% of cows with a maximum of 3 positive quarters. *Mycoplasma* spp. and *Enterococcus* spp.*+ L. lactis* ssp. *lactis* were detected in 6 and 27 cows, respectively (Table 3), mostly in 1 quarter for *Mycoplasma* spp. and in almost 2 quarters for *Enterococcus* spp.*+ L. lactis* ssp. *lactis* (Fig. 2).

### Correlations

Table 4 summarizes the Pearson's correlation coefficients calculated within each pathogen group between the milk udder health indicators (namely SCS, DSCC, and DSCS) and milk traits, i.e., milk yield and solids content. Overall, SCS was strongly positively correlated with both DSCC and DSCS and inversely with milk yield and lactose, similarly to DSCS. The SCS and DSCS were not significantly or positively correlated with fat and protein content. DSCC was not significantly associated with milk yield and lactose.

**Table 4:** Pearson’s correlations^a^ (*P* ≤ 0.05) between udder health-related milk traits^b^ within each pathogen group^c^

Trait	SCS	DSCC, %	DSCS	SCS	DSCC, %	DSCS
	**Major, ≤ 2 quarters**			**Major, ≥ 3 quarters**		
**DSCC, %**	0.65			0.64		
DSCS	0.99	0.73		0.99	0.73	
Milk yield, kg/d	-0.19	*ns*	-0.17	-0.25	*ns*	-0.23
Fat, %	*ns*	*ns*	*ns*	*ns*	*ns*	*ns*
Protein, %	*ns*	-0.13	*ns*	*ns*	*ns*	*ns*
Casein, %	*ns*	-0.13	*ns*	*ns*	*ns*	*ns*
Casein index	*ns*	*ns*	*ns*	-0.21	-0.25	-0.22
Lactose, %	-0.34	*ns*	-0.31	-0.55	-0.23	-0.53
	**Minor, ≤ 2 quarters**			**Minor, ≥ 3 quarters**		
DSCC, %	0.54			0.58		
DSCS	0.98	0.69		0.99	0.68	
Milk yield, kg/d	-0.38	*ns*	-0.35	-0.41	*ns*	-0.37
Fat, %	0.21	*ns*	0.18	0.35	*ns*	0.33
Protein, %	0.41	*ns*	0.35	0.47	*ns*	0.44
Casein, %	0.37	*ns*	0.32	0.44	*ns*	0.41
Casein index	-0.04	*ns*	-0.02	*ns*	*ns*	*ns*
Lactose, %	-0.45	*ns*	-0.39	-0.52	*ns*	-0.48
	**Other, ≤ 2 quarters**			**Other, ≥ 3 quarters**		
DSCC, %	0.66			0.56		
DSCS	0.99	0.76		0.98	0.69	
Milk yield, kg/d	-0.49	*ns*	-0.44	-0.46	*ns*	-0.46
Fat, %	*ns*	*ns*	*ns*	*ns*	*ns*	*ns*
Protein, %	0.40	*ns*	0.34	*ns*	*ns*	*ns*
Casein, %	0.40	*ns*	*ns*	*ns*	*ns*	*ns*
Casein index	*ns*	*ns*	*ns*	-0.17	*ns*	*ns*
Lactose, %	-0.33	*ns*	-0.27	-0.48	*ns*	-0.43
	Pathogens absent					
DSCC, %	0.49					
DSCS	0.98	0.65				
Milk yield, kg/d	-0.39	*ns*	-0.35			
Fat, %	0.13	*ns*	0.12			
Protein, %	0.30	*ns*	0.26			
Casein, %	0.29	*ns*	0.25			
Casein index	*ns*	*ns*	*ns*			
Lactose, %	-0.30	0.16	-0.23			

^a^*ns* Not significant

^b^SCS ﻿Somatic cell score, calculated as SCS = 3 + log_2_(SCC/100,000), where SCC is somatic cell count (cells/mL), *DSCC* Polymorphonuclear neutrophils and lymphocytes (%) out of the total SCC, *DSCS* Differential somatic cell score, calculated as DSCS = 3 + log_2_(DSCC_N_/100,000), where DSCC_N_ is the combined number of polymorphonuclear neutrophils and lymphocytes in milk (cells/mL)

^c^Description of groups is provided in Tables 1 and 2

**Fig. 3:**
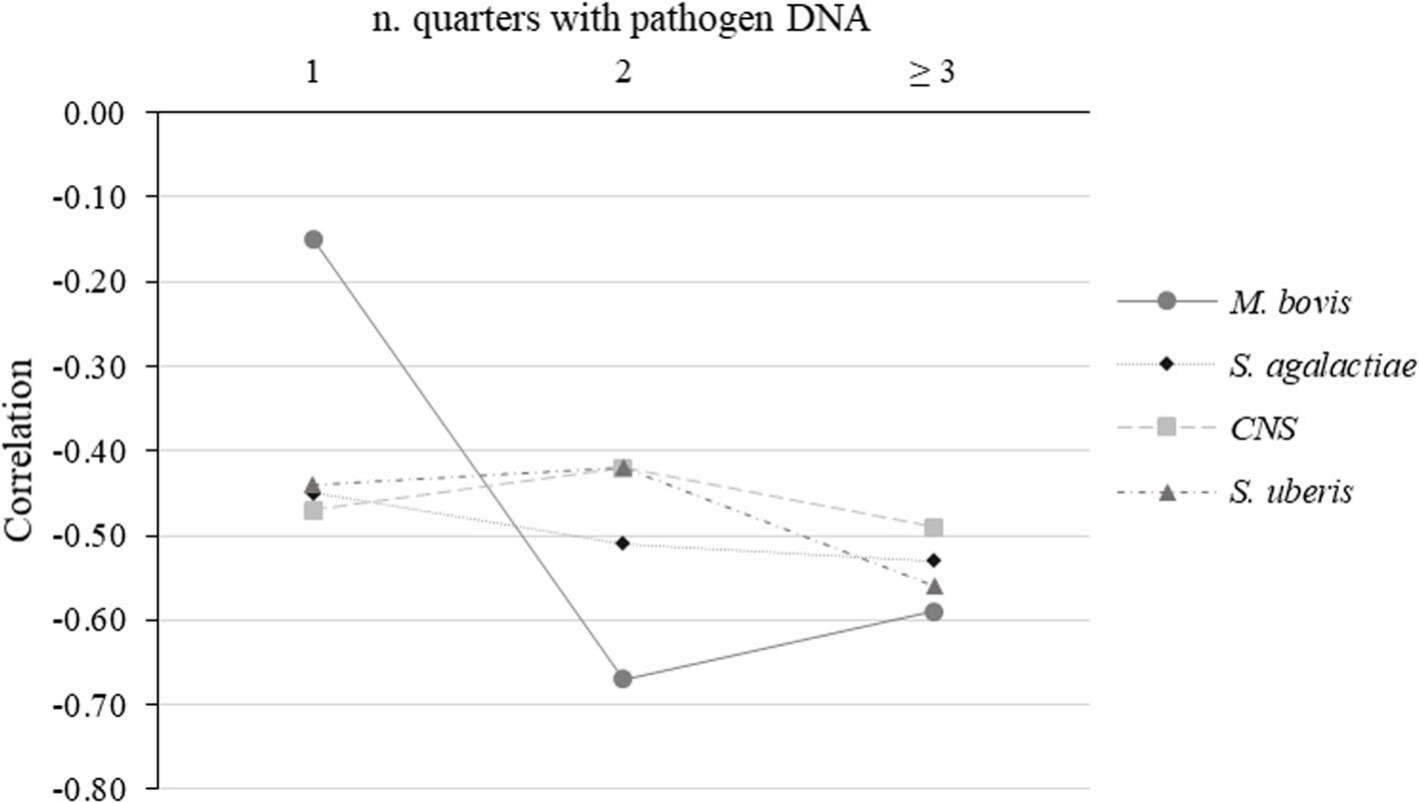
Pearson’s correlations (*P*≤ 0.05) between lactose content and SCS in cows with pathogen DNA^1^ isolated in 1, 2, or ≥ 3 quarters.﻿ ^1^*S. agalactiae*, *S.uberis*, CNS (coagulase-negative staphylococci), and *M. bovis*

Regarding cows with ≥ 3 quarters with pathogen DNA, in most cases the correlation was stronger in magnitude compared to cows free of any pathogens. Lactose content, for example, was negatively correlated with SCS also in cows with no pathogens detected, but the association was stronger when 3 or 4 quarters had pathogen DNA (Table 4). For specific pathogens (*S. agalactiae*, *S. uberis*, CNS, and *M. bovis*; Fig. 3) the correlation between lactose content and SCS was negatively stronger with more quarters with pathogen DNA. The correlation between milk yield and SCS differed according to pathogen group and number of quarters with pathogen DNA. To better explore the lactose trend, its correlations with SCS were assessed in cows with 1, 2, ≥ 3 quarters with the following microorganisms: *S. agalactiae, S. uberis, CNS,* and *M. bovis*. In this study only the most isolated microorganisms were considered (Table 3; Fig. 2). Figure 2 depicts how the magnitude of correlation increased moving from 1 (26 cows) to 2 (6 cows) and 3 (28 cows) infected quarters in the case of *M. bovis*. A lesser sharp increase in magnitude was observed for *S. agalactiae*, while no linear pattern was detected for CNS and *S. uberis*.

### Presence vs. absence of pathogen

**Fig. 4:**
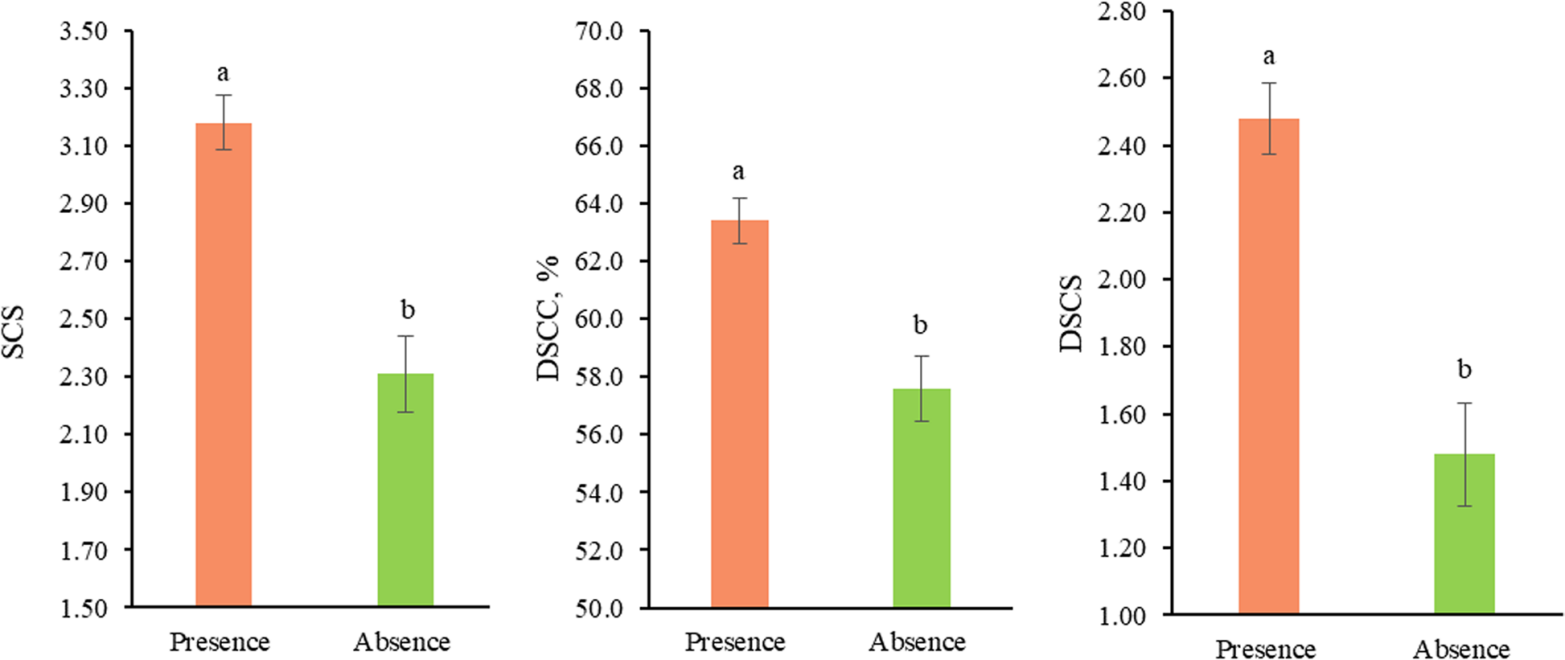
Least squares means^1^ of udder health-related traits for the fixed effect of pathogen DNA. ﻿^1^ Values with different superscripts within trait differ significantly (*P *≤ 0.05)

The presence of pathogen DNA significantly affected the udder health indicators present in milk. In fact, the effect in (Eq. 1) was able to explain part of the variability of SCS, DSCC, and DSCS (*P* ≤ 0.05). In particular, SCS ranged from 2.31 ± 0.13 in cows without pathogen DNA to 3.18 ± 0.09 in those with pathogen DNA in at least one quarter (Fig. 4). These two SCS values correspond to SCC of approximately 61,980 and 113,280 cells/mL, respectively. The same trend occurred for DSCC, which moved from 57.60 ± 1.12 to 63.40 ± 0.78, and for DSCS, from 1.48 ± 0.15 to 2.48 ± 0.11 (Fig. 4).

### Effect of pathogen group

The pathogen group (Eq. 2) was statistically significant in explaining the variability of SCS, DSCC, DSCS, lactose content, and casein index (*P* ≤ 0.05). The other investigated traits (i.e., milk yield, and fat, protein, and casein contents) were not affected. Cows free of pathogens had the lowest SCS (2.29 ± 0.13), DSCC (57.50 ± 1.12%), and DSCS (1.46 ± 0.15; Table 5). Within the pathogen groups (i.e., major, minor, and other), cows with pathogen DNA present in ≥ 3 quarters had greater SCS, DSCC, and DSCS than cows with pathogen DNA in ≤ 2 quarters. For all the udder health related traits, the greatest/worse LSM was estimated for the more extreme scenario, i.e., cows with major pathogens in ≥ 3 quarters (Table 5). Regarding the contrast estimates (Table 6), cows free from pathogens had lower SCS, DSCC, and DSCS (*P* < 0.001) than those with pathogen DNA in at least one quarter. At the same time, however, cows with DNA of minor pathogen group had lower SCS, DSCC, and DSCS compared to those with major and other group pathogen (Table 6). For example, milk SCS was 2.29, 3.09, and 3.68 in the following groups: absent, minor in at least 3 quarters, and major in at least 3 quarters (Table 5). These values correspond to SCC of approximately 61,130, 106,400, and 160,200 cells/mL, respectively.

**Table 5:** Least squares means^1^ (standard error) of milk traits for the fixed effect of pathogen group^2^

Trait^3^	Absent pathogens	Major pathogens		Minor pathogens		Other pathogens	
		≤ 2 quarters	≥ 3 quarters	≤ 2 quarters	≥ 3 quarters	≤ 2 quarters	≥ 3 quarters
SCS	2.29 (0.13)^c^	3.23 (0.14)^ab^	3.68 (0.19)^a^	2.73 (0.16)^bc^	3.09 (0.24)^abc^	3.12 (0.31)^abc^	3.59 (0.38)^ab^
DSCC, %	57.50 (1.12)^b^	64.70 (1.18)^a^	65.00 (1.63)^a^	60.40 (1.38)^ab^	62.40 (2.07)^ab^	63.20 (2.64)^ab^	64.40 (3.16)^ab^
DSCS	1.46 (0.15)^c^	2.58 (0.16)^ab^	3.01 (0.22)^a^	1.96 (0.19)^bc^	2.36 (0.28)^abc^	2.42 (0.36)^abc^	2.90 (0.43)^ab^
Milk yield, kg/d	25.90 (0.47)	24.50 (1.13)	24.90 (0.70)	25.30 (0.59)	24.90 (0.70)	24.50 (1.13)	26.60 (1.35)
Fat, %	3.84 (0.05)	3.90 (0.06)	3.79 (0.08)	3.93 (0.06)	3.88 (0.10)	4.10 (0.124)	4.06 (0.15)
Protein, %	3.63 (0.02)	3.66 (0.02)	3.64 (0.03)	3.64 (0.03)	3.63 (0.04)	3.72 (0.06)	3.71 (0.07)
Casein, %	2.90 (0.02)	2.92 (0.02)	2.88 (0.03)	2.90 (0.03)	2.89 (0.04)	2.98 (0.05)	2.97 (0.06)
Casein index	0.798 (0.001)^a^	0.797 (0.001)^ab^	0.791 (0.001)^b^	0.796 (0.001)^ab^	0.796 (0.002)^ab^	0.798 (0.002)^ab^	0.800 (0.001)^ab^
Lactose, %	4.77 (0.01)^a^	4.77 (0.01)^a^	4.67 (0.02)^b^	4.76 (0.01)^a^	4.73 (0.02)^ab^	4.80 (0.03)^a^	4.80 (0.04)^a^

^1^Values with different superscripts within trait differ significantly (*P* ≤ 0.05)

^2^Description of groups is provided in Tables 1 and 2

^3^*SCS* Somatic cell score, calculated as SCS = 3 + log_2_(SCC/100,000), where SCC is somatic cell count (cells/mL), *DSCC* Polymorphonuclear neutrophils and lymphocytes (%) out of the total SCC, *DSCS* Differential somatic cell score, calculated as DSCS = 3 + log_2_(DSCC_N_/100,000), where DSCC_N_ is the combined number of polymorphonuclear neutrophils and lymphocytes in milk (cells/mL)

**Table 6:** Contrast estimates^a^ (standard error) for milk traits within pathogens group^b^

Trait^c^	Absent vs. present	Absent vs. minor	Absent vs. major	Absent vs. other	Minor vs. major	Minor vs. other	Major vs. other
SCS	-5.69 (1.00)***	-1.23 (0.39)**	-2.33 (0.35)***	-1.23 (0.39)**	-1.10 (0.37)**	-0.89 (0.57)	0.21 (0.54)
DSCC, %	-34.95 (8.53)***	-7.78 (3.32)*	-14.63 (2.93)***	-7.78 (3.32)*	-6.84 (3.13)*	-4.75 (4.81)	2.09 (4.54)
DSCS	-6.47 (1.16)***	-1.40 (0.45)**	-2.67 (0.40)***	-1.40 (0.45)**	-1.27 (0.42)**	-0.99 (0.65)	0.28 (0.62)
Milk yield, kg/d	4.54 (3.60)	1.60 (1.39)	2.32 (1.24)	1.60 (1.39)	0.72 (1.33)	-0.96 (2.05)	-1.68 (1.94)
Fat, %	-0.63 (0.40)	-0.14 (0.16)	-0.019 (0.14)	-0.14 (0.16)	0.12 (0.15)	-0.34 (0.23)	-0.46 (0.21)*
Protein, %	-0.21 (0.18)	-0.002 (0.07)	-0.03 (0.06)	-0.002 (0.07)	-0.03 (0.06)	-0.17 (0.10)	-0.14 (0.10)
Casein, %	-0.13 (0.16)	0.01 (0.06)	-0.001 (0.05)	0.01 (0.06)	-0.01 (0.06)	-0.16 (0.09)	-0.15 (0.08)
Casein index	0.009 (0.008)	0.004 (0.003)	0.01 (0.00)**	0.004 (0.003)	0.004 (0.003)	-0.007 (0.004)	-0.010 (0.004)
Lactose, %	0.08 (0.10)	0.04 (0.04)	0.10 (0.04)**	0.04 (0.04)	0.06 (0.04)	-0.11 (0.06)	-0.16 (0.06)

^a^***P ≤ 0.001, **P ≤ 0.01, *P ≤ 0.05

^b^Description of groups is provided in Table 2

^c^*SCS* Somatic cell score, calculated as SCS = 3 + log_2_(SCC/100,000), where SCC is somatic cell count (cells/mL), *DSCC* Polymorphonuclear neutrophils and lymphocytes (%) out of the total SCC, *DSCS* Differential somatic cell score, calculated as DSCS = 3 + log_2_(DSCC_N_/100,000), where DSCC_N_ is the combined number of polymorphonuclear neutrophils and lymphocytes in milk (cells/mL)

Cows with major pathogens in ≥ 3 quarters had the lowest casein index (0.792 ± 0.001; Table 5). The greatest LSM was estimated for cows with no pathogen DNA. In terms of lactose content, the lowest estimate was for cows with major pathogens in ≥ 3 quarters (4.67 ± 0.02%). The lactose content in the other classes was statistically similar to that of the cows with no DNA pathogen (4.77 ± 0.01%; Table 5). As regards the contrast estimates (Table 6), cows without pathogen DNA in any quarter had greater lactose content (0.10 ± 0.04; *P* < 0.001) and casein index (0.01 ± 0.00; *P* < 0.001) than cow with DNA of major pathogens.

Although milk yield did not significantly differ among the pathogen groups, numerically, cows free of any DNA yielded more milk (+ 4.54 kg) than those with pathogen(s) detected. Cows with DNA of major pathogens had lower fat content (-0.46 ± 0.21; *P* < 0.05) than the others.

## Discussion

### Prevalence of pathogens

The present study dealt with 9 farms located in the Northern Italy, where all clinically healthy cows were sampled. However, DNA of at least one target pathogen was isolated in at least one quarter in all the herds (Table 2). Twelve popular mastitis-causing species were detected via the qPCR commercial kit ‘Mastitis 4BDF’, even though microorganisms that can potentially cause mastitis are more numerous [26].

In this study about 27.2% of cows had the DNA of at least 1 of the twelve pathogens. In particular, the average number of quarters with one or more pathogens isolated ranged from 1.0 to 2.8 per cow (Table 3). These results seem more favourable than those obtained by Piepers et al. [27] who reported that approximately 40% of the cows had at least 1 infected quarter in Flemish dairy herds. These authors, however, performed bacterial culture on more than 170,000 quarter milk samples collected in 1,087 cross-sectional dairy herd screenings performed in three consecutive years.

The most detected DNA was the CNS (Fig. 2). This finding aligns with results of several authors who performed bacterial cultures [28, 29] or qPCR for the analysis of either composite [23] or quarter milk [30]. Different prevalence has been detected depending on whether clinically healthy cows or with suspected mastitis were sampled. However, CNS are a group of opportunistic pathogens that commonly lay on the teat skin and some of the positive samples could have resulted from contamination (e.g., teat canal) during milk ejection rather than from a real infection of the gland cistern. In fact, CNS cause clinical or subclinical inflammation only when certain conditions favor their colonization in the udder, e.g., poor milking hygiene, inadequate teat disinfection, disturbances/stressors, and compromised immune response [31]. In the present study, the prevalence of *S. uberis* (Table 3) at cow level was greater than in others [26, 29], but in line with that reported by Vakkamäki et al. [30]. However, these authors detected pathogen DNA by using the ‘PathoProof Mastitis PCR Complete-12’ assay in quarter milk samples from more than 90,000 Finnish cows with a diagnosis of mastitis (4,725 herds). Schwarz et al. [23] took composite milk in aseptic conditions from 576 cows in 11 Canadian herds and reported a lower prevalence (4.5%) for *S. uberis* determined through bacteriological culture. *S. uberis* is a ubiquitous microorganism that causes clinical and subclinical mastitis in dairy cows. It is commonly isolated from the environment and barn bedding material represents the main site of infection and contamination [32]. In this study (Table 3), *S. agalactiae* was detected with similar or higher prevalence than other studies who analysed milk samples from cows with mastitis [30, 33]. In particular, Kalmus et al. [33] used the same qPCR assay adopted in the current study to analyze quarter milk samples of 263 Estonian cows and reported a prevalence of 5.3%. Vakkamäki et al. [30] reported a very low prevalence (0.4%).

The prevalence of other major pathogens (i.e., *S. aureus, S.dysgalactiae*, *E. coli*, and *Klebsiella* spp.) is in line with other studies that investigated microorganism prevalence in clinically healthy cows, i.e., Kurban et al. [26] and Suntinger et al. [29]. The first reported species-specific prevalence using the MALDI-TOF in quarter milk samples from 50,429 healthy cows, whereas the second performed bacteriological analyses in more than 6,800 quarter milk samples of Austrian Fleckvieh cows in 248 farms.

Regarding other groups, the *M. bovis* DNA was frequently detected in this study. *M. bovis* had the greatest average number of quarters (2.8) with pathogen DNA among the target microorganisms (Table 3). *M. bovis* is the most spread species of *Mycoplasma* in bovine milk, is a contagious microorganism, and typically causes subclinical or mild clinical IMI, which can often progress to chronic mastitis [34]. Few studies have considered these microorganisms so far because of the bacterial cultures: in fact, *Mycoplasma* is difficult to detect due to low sensitivity and long incubation periods [15]. The prevalence of *Enterococcus + L. lactis* ssp. *lactis* in this study was low, in agreement with other reports presenting microorganism prevalence in healthy cows [23, 29]. Finally, *Prototheca* spp. affected on average 1.5 quarters, when present (Table 3). This family includes algae organisms that can invade the udder tissue and cause mastitis [35]. Vakkamäki et al. [30] isolated this microorganism with a similar prevalence (1.3% at cow level; Table 3) in 90,000 dairy cows with mastitis from 4,725 Finnish herds. Generally, *Prothoteca* spp. are isolated in the environment in different substrates such as bedding, barn walls, feed, and drinking water [36].

### Correlations

Associations between all the traits have been calculated for cows with major, minor, and other pathogens and, at the same time, separately for cows with ≤ 2 and ≥ 3 positive quarters (Table 4). The same were also calculated for cows with all quarters free of pathogens (Table 4). Overall, coefficients indicate that the presence of at least 1 quarter with a pathogen DNA affects the magnitude of correlations, especially for lactose content. Lactose was more strongly correlated with SCS and DSCC when at least 1 microorganism was present in at least 1 quarter; the strongest association (-0.55) was observed with SCS in cows with ≥ 3 quarters infected with major microorganisms and the weakest (-0.30) when the microorganisms were absent. In general, it was observed that this correlation increases with the number of quarters with pathogen DNA. Costa et al. [37] demonstrated that lactose content can be a valid indicator of mastitis in dairy cows, due to its association with other markers of mastits (SCC and electrical conductivity), exploitable heritability, and availability on a large scale as part of the DHI testing. Indirect correlations between lactose and both SCS and DSCS were expected, but for the first time this manuscript provides evidence of the different magnitude according to the number of quarters infected and type of microorganisms involved (Fig. 3). In the case of *M. bovis*, the correlation between lactose and SCS increased dramatically in magnitude when comparing cows with 1 quarter infected with cows with more quarters infected (Fig. 3). The association was, in fact, around -0.15 in the first case and nearby -0.65 in the second (Fig. 3).

A significant correlation of lactose with DSCC was observed in only two cases (Table 4), i.e., when considering cows with ≥ 3 quarters infected with major microorganisms (-0.23) and when considering the absence of pathogens (0.16). The two coefficients had opposite signs, whereas the correlations between lactose and DSCS were always negative, suggesting that using raw values of DSCC instead of its log-transformation, may lead to biased conclusions. As an example, Bobbo et al. [38] estimated a correlation of almost zero (-0.05) between lactose and DSCC in the Holstein breed, while the correlation with SCS was stronger (-0.26) and similar to that of this study. Using DSCC (in classes) as a fixed effect in the analysis of variance, Pegolo et al. [39] reported that not only milk lactose content but also milk yield and casein index tend to linearly increase as the DSCC increases and concluded that there is a need to better study the mechanisms underpinning the high milk lactose in presence of high DSCC.

Milk yield was uncorrelated with DSCC, but was negatively associated with both SCS and DSCS in all cases. Contrary to expectations, the weakest association was estimated in cows with ≤ 2 quarters affected by major pathogen(s) and not in cows with all quarters free of microorganisms.

Except for a few cases, no significant correlations were observed between DSCC and milk composition traits, regardless of the number of quarters with pathogen DNA and type of microorganisms involved. The correlations of casein index were in general not significant, except for quarters with ≥ 2 quarters with major pathogens (Table 4). In this specific case, the casein index was negatively correlated with SCS (-0.21), DSCC (-0.25), and DSCS (-0.22). Fat content was basically not – or very weakly and positively – correlated with the udder health traits.

### Effect of pathogen presence on milk traits

As previously mentioned, presence of pathogen DNA in at least one quarter was not indicative of a truly infected cistern, as some DNA can be found in the teat canal. Moreover, the qPCR-based detection allows for the isolation of non-living microorganisms, i.e., including pathogens that were successfully eliminated by the cow’s immune system before multiplication.

Despite all the cows did not present clinical signs of mastitis and were overall clinically healthy, findings reveal that the presence of pathogen in their milk can often impair milk solids composition and udder health-related traits. Cows with at least 1 quarter with pathogen DNA had higher SCS, DSCC, and DSCS in their pooled milk compared to cows free of pathogen DNA (Tables 5 and 6). The type of microorganisms play a crucial role in the inflammatory response and in the subsequent physiological increase in SCC. The peak of SCC and its persistence in milk are pathogen-specific in dairy cows [40]. Schwarz et al. [23] performed qPCR on composite milk samples and reported that cows with major pathogen DNA in the udder had generally the greatest SCS and DSCC; on the contrary, cows with DNA of minor and other pathogens had similar SCS and DSCC of animals free of pathogens. The same was observed by Kirkeby et al. [19], who performed bacterial cultures on milk samples at quarter level and qPCR on composite milk samples from Danish cows.

Usability and interpretation of DSCC in cattle is a matter of debate. Some authors stated that the extent to which the diagnostic accuracy increases with DSCC depends on several factors. For instance, changes in the pattern of SCC, DSCC, and other milk traits differ according to the number of quarters infected as well as the type of pathogen involved [40, 41]. It is well known that different microorganisms induce different immune responses in the mammary gland [42], but the specific effect of major and minor pathogens on both SCC and DSCC is far to be considered totally understood and needs more investigation.

Within the pathogen group, cows with pathogen DNA in ≤ 2 quarters had lower values of udder health-related traits (Table 5), which can be attributed to attenuation. In this study the average number of quarters with pathogen DNA per cow varied according to the type of microorganisms involved (Table 3). It is important to note that gross composition here refers to the composite milk (mixture of functional quarters), thus when the number of positive quarters is small (e.g., 1) the overall milk composition and udder health-related traits may be less influenced compared to situations where the number of positive quarters is ≥ 3.

In this study, the presence of pathogen DNA affected udder health-related traits but not milk yield. This can be explained easily, because (i) the milk yield data available was pooled, not at quarter level, and (ii) healthy lactating cows were sampled, so no clinical mastitis with severe inflammation process were present. It is known that the most productive cows are those most susceptible to IMI due to the great stress to which they are exposed [43]. In contrast, cows with signs of clinical mastitis present altered milk yield [28, 44]. Botari et al. [44] reported *S. aureus* subclinical mastitis at quarter level negatively influenced daily production; only the quarter with the IMI is expected to be responsible for milk yield decrease. The literature has also reported pathogen-specific patterns of milk production losses caused by clinical mastitis [28]. In terms of milk yield loss, literature says that the most harmful pathogen causing mastitis is *E. coli*. Other major pathogens (e.g., *S. aureus* and *Klebsiella* spp.) negatively affect milk production (at quarter level) too, but generally to a lesser extent [45, 46]. Tomazi et al. [47] and Valckenier et al. [48] reported that subclinical mastitis caused by CNS can increase the SCC but – as most the subclinical inflammations - had no evident effect on milk yield.

As described in other studies [49, 50], the presence of microorganisms in the udder does not significantly affect the fat, protein, and casein content of milk (Table 5). In the present study, the casein index was lower (0.791) in cows with major pathogens compared to cows with no pathogens (0.798) in line with Bobbo et al. [50]. A decrease in the casein index is due to a reduced proteins synthesis in the mammary gland (such as α-casein, β-casein, α-lactalbumin, and β-lactoglobulin) followed by an increase in third protein fractions transferred from the blood, such as serum albumin [51]. As described by Boutinaud et al. [52], a reduction in milk protein content is expected in presence of mammary tissue inflammation.

As previously discussed, authors have already reported a decrease in lactose content in milk from cows with IMI or clinical/subclinical mastitis [6, 37]. In this study, although cows free of clinical mastitis were involved, a significant effect of pathogens was observed (Table 5). The LSM are in line with expectations, demonstrating that this milk component reduces in presence of one or more pathogens. In cows with IMI and/or high SCC, the drop in milk lactose is due to leakage caused by the compromised alveolar epithelial integrity, but there may be cases where latent and/or past mastitis events have permanently altered the epithelial integrity and thus the lactose content of a cow [6, 37, 53].

### Practical considerations

In terms of limitations, authors acknowledge that in this study milk yield and composition was available at cow rather than quarter level. Further studies should evaluate how the presence of pathogen DNA affects the quarter-level milk yield and composition to establish the deterioration of the performance of the infected quarter(s) solely. The design adopted in this study mirrors real in-field situations where collecting representative milk samples for each individual quarter is not feasible on a routine basis. Composite milk data obtained from official DHI still are official and subjected to standardization. On the contrary, obtaining standardized milk data for individual quarters on a large scale is challenging, especially if samples have to be collected in sterile conditions. Having the possibility to analyse bulk milk in the herd or individual milk with qPCR or on-farm culture kits may represent a potential screening tool of interest for dairy farmers for decision-making. In the era of antimicrobial resistance and treatments restriction, accurate identification of IMI and subclinical forms of mastitis is fundamental for a smart selective dry-cow therapy and pathogens eradication. Testing the quarter milk of late-lactation cows close to the dry-off, for instance, may represent a valid opportunity to disclose quarters/cows needing a treatment, to identify cows that can contaminate the others, and to adopt the most appropriate/target selective dry-cow therapy protocol.

Another point to consider when interpreting the findings of this research is the fraction of non-living microorganisms. The qPCR assay, in fact, detects DNA of both living and non-living pathogens, meaning that pathogens already inactivated or killed by the cow’s immune system but potresent in the teat canal or cistern are identified too. This issue can be overcome with the MALDI-TOF integration into diagnostic protocols which accurately identifies both target and non-target pathogens through analysis of their mass spectra [16] and allows the recognition just of the living microrganisms. Currently, the MALDI-TOF databases do not include all milk-associated bacterial species [54].

## Conclusion

Clinically healthy cows may present microorganisms in their milk due to presence of pathgens in the mammary gland cistern (proper infection) and/or in the teat canal. In this study, the pathogen group (major, minor, and other) and the number of quarters infected significantly affected the cows’ milk SCS, DSCC, and DSCS. The presence of microorganisms in milk affected the lactose content and the casein index, with the lowest estimate for cows with major pathogens isolated in ≥ 3 quarters. Although data refer to healthy cows, results indicate that SCS and lactose content - both indicators of mastitis - undergo different changes in milk according to the pathogen group involved and number of infected quarters. Certainly, findings should be confirmed using quarter milk composition traits rather than composite (pooled) traits. In the era of antimicrobial resistance and treatments restriction, accurate identification of pathogen(s) causing subclinical forms of mastitis is fundamental for the definition of smart and tailored strategies such as the selection of the most appropriate selective dry-cow therapy protocol. Work has still to be done in the dairy sector to be efficient in the early detection, since most of the diagnostic material commercially available for mastitis is quite costly and designed for the detection of preselected pathogens exclusively thereby limiting the detection of third and relatively unknown microorganisms potentially dangerous for the udder health.

## Data Availability

Data used to generate results of this study is available upon request from the corresponding author.

## Abbreviations

IMI: Intramammary infection
PMN: Polymorphonuclear neutrophils
SCC: Somatic cell count
DSCC: Differential somatic cell count
qPCR: Quantitative PCR
STER: Sterile conditions
DHI: Dairy herd improvement
CNS: Coagulase-negative staphylococci
NAS: Non-*aureus* staphylococci
SCS: Somatic cell score
DSCS: Differential somatic cell score
LSM: Least squares means
